# Bunk space requirements for growing beef cattle limit-fed a high-energy corn and corn co-product diet

**DOI:** 10.1093/tas/txac096

**Published:** 2022-07-27

**Authors:** Zachary M Duncan, Zachary L DeBord, Madison G Pflughoeft, Kyler J Suhr, William R Hollenbeck, Anthony J Tarpoff, K C Olson, Dale A Blasi

**Affiliations:** Department of Animal Science and Industry, Kansas State University, Manhattan, KS 66506, USA; Department of Animal Science and Industry, Kansas State University, Manhattan, KS 66506, USA; Department of Animal Science and Industry, Kansas State University, Manhattan, KS 66506, USA; Department of Animal Science and Industry, Kansas State University, Manhattan, KS 66506, USA; Department of Animal Science and Industry, Kansas State University, Manhattan, KS 66506, USA; Department of Animal Science and Industry, Kansas State University, Manhattan, KS 66506, USA; Department of Animal Science and Industry, Kansas State University, Manhattan, KS 66506, USA; Department of Animal Science and Industry, Kansas State University, Manhattan, KS 66506, USA

**Keywords:** bunk, grazing, growing calves, limit-feeding

## Abstract

Bunk requirements for optimal growth performance of growing calves limit-fed high-energy corn and corn co-product diets have not been widely evaluated. Three-hundred eighty-five crossbred steers (initial body weight = 215 ± 25 kg) were purchased in Texas, transported to the Kansas State Beef Stocker Unit, and weighed at arrival. Steers were stratified by body weight and randomly assigned to 1 of 28 pens containing 12 to 14 head. Within block, pens were randomly assigned to one of four bunk allotment treatments: 25.4, 38.1, 50.8, or 63.5 cm of bunk per head for a 58-d receiving period. Calves were fed at 0700 h once daily at 1.8% of bodyweight (dry matter basis) from February 2 to March 13, 2021; thereafter the daily feed allotment was increased to 2.0% of bodyweight. The diet contained (dry matter basis) 39.5% dry-rolled corn, 7.5% supplement, 40% wet corn gluten feed, and 13% prairie hay. Steers were individually weighed on days 29 and 58 and pen weights were measured weekly to determine feed offered for the following week. Body weights on days 29 and 58, dry matter intake, or gain-to-feed ratio during the receiving period did not differ (*P* ≥ 0.34) between treatments. During the first 29 d, average daily gain (ADG) increased linearly as bunk space increased (*P* = 0.03); however, no treatment effects were observed thereafter. In addition, ADG standard deviation from days 0 to 29 responded quadratically (*P* = 0.05) where ADG standard deviation tended to be greater in the 38.1-cm allotment and was greater in the 50.8-cm allotment compared to the 25.4-cm allotment (*P* = 0.07 and *P* = 0.04, respectively). Bunk score tended to decrease linearly as bunk allotment decreased (*P* = 0.06). Following the receiving period, steers were blocked by bunk treatment and randomly assigned to 1 of 18 pastures. Steers were grazed for 90-d from May to August at a targeted stocking density of 280 kg live-weight ˖ ha^–1^. During the grazing season, ADG increased linearly with reduced (*P* < 0.01) bunk allotment; however, body weights did not differ (*P* = 0.91) between bunk treatments at the completion of the grazing period. In addition, overall total body weight gains and ADG from the receiving and grazing periods did not differ (*P* > 0.57) between bunk treatments. We interpreted our data to suggest that bunk space allotments of 25.4 to 63.5 cm per head had minimal impact on growth performance during a 58-d receiving period and did not affect final body weights following a 90-d grazing season.

## INTRODUCTION

Limit-fed high energy diets can improve feed efficiency in growing calves compared with traditional high-roughage diets fed ad libitum (Wagner et al., 1990; Spore et al., 2019). One concern associated with limit feeding is that bunk requirements may need to be increased when feed is restricted in order to ensure that all calves can eat simultaneously. Lake (1986) reported bunk allotments of 23 cm per head allowed approximately 55% of calves to eat at once while 30 cm of bunk allowed approximately 75% of calves to eat at once. Current recommendations for 180–380 kg beef calves fed once daily are 45.7–55.9 cm of bunk per calf (FASS, 2020).

Feed bunks represent a significant investment for cattle feeders and currently cost up to $82–98 per linear meter (Kammel and Halfman, 2015). Although limit-fed diets can improve feed efficiency in growing calves, the cost of purchasing additional bunk may outweigh the benefits in improved performance. Determining bunk allotment required for limit-fed growing calves is necessary to optimize growth performance and maximize pen capacity; therefore, the objective of this experiment was to evaluate the effects of bunk-space allotment on growth performance of growing calves limit-fed a high-energy corn and corn co-product diet during a 58-d receiving period. An additional objective was to determine if bunk-space allotment during the receiving period impacted subsequent growth performance during a 90-d grazing season in the Kansas Flint Hills.

## MATERIALS AND METHODS

The Kansas State University Institutional Animal Care and Use Committee reviewed and approved all animal handling and animal care practices used in our experiment. All animal procedures were conducted in accordance with the Guide for the Care and Use of Animals in Agricultural Research and Teaching (FASS, 2020).

A total of 385 crossbred steers (initial body weight: 215 ± 25 kg) were purchased in Texas and transported to the Kansas State Beef Stocker Unit. The first two truckloads of cattle were received on 2 February and the second two truckloads were received on 2 March. Steers were arranged in a randomized block design to determine the effects of bunk-space allotment on growth performance of growing beef cattle limit-fed a high-energy corn and corn co-product diet. Calves were blocked by arrival date (2), stratified by individual arrival weight within block, and assigned to pens containing 12–14 head. Within block, pens were randomly assigned to one of four treatments which resulted in 7 pens per treatment for a total of 28 pens. Soil surfaced pens equal in size (9.1 × 15.2 m) contained a fenceline feed bunk, a 3.6-m concrete apron, and an individual automatic waterer. Bunk length was adjusted to allow 25.4, 38.1, 50.8, or 63.5 cm of bunk per calf. Panels were fastened along each fenceline bunk to restrict bunk allotment without altering pen size.

Upon arrival, steers were individually restrained using a hydraulic squeeze chute (Silencer, Moly Manufacturing Inc., Lorraine, KS), bodyweight (BW) was recorded, and a visual identification tag was applied. Subsequently, animals were randomly assigned to pens containing 12–14 steers and provided prairie hay and ad libitum access to water overnight. The following morning (day 0), steers were individually weighed, vaccinated for viral respiratory (Vista Once SQ; Merck Animal Health, Kenilworth, NJ) and clostridial (Vison 7 with Spur; Merck Animal Heath, Kenilworth, NJ) pathogens and treated for internal (Valbazen, Zoetis; Parisippany, NJ) and external (Stand Guard, Elanco; Greenfield, Indiana) parasites.

Individual BW were measured on days 0, 29, and 58. In addition, pen weights were measured weekly (days 0, 14, 21, 28, 35, 42, 49, and 56) using a pen scale (Rice Lake Weighing Systems; Rice Lake, WI) and were used to calculate the feed delivered for the following week. Steers were fed once daily at 0700 h using a Roto-Mix feed wagon (Model #414-14B; Roto-Mix, Dodge City, KS). The experimental diet (Table 1) was offered at 1.8% of BW (dry matter basis) from 2 February to 13 March, 2021; thereafter, the daily feed allotment was increased to 2.0% of BW (dry matter basis). Individual feed ingredient samples were collected weekly and immediately frozen at –20 °C. At the completion of the experiment, feed ingredient samples were composited and sent to a commercial laboratory for nutrient analysis (SDK Laboratories; Hutchinson, KS).

**Table 1.** Composition of experimental diet

Item,	
Ingredient, % of dry matter	
Dry-rolled corn	39.5
Supplement^1^	7.5
Wet corn gluten feed^2^	40.0
Prairie hay	13.0
Nutrient Composition, % of dry matter^3^	
Dry matter	75.3
Organic matter	94.1
Crude protein	14.9
Neutral detergent fiber	24.7
Acid detergent fiber	9.4
Calculated Composition^4^, Mcal/kg	
NEm	1.95
NEg	1.31

Supplement pellet formulated to contain (dry matter basis) 8.4% Ca, 5% NaCl, and 360 mg/kg monensin. Supplement ingredients: 72.15% wheat middlings, 22.0% calcium carbonate, 5% NaCl, 0.35% soybean oil, 0.18% Rumensin 90 (Elanco, Greedfield, IN), 0.11% zinc sulfate, 0.08% manganese (Mn) sulfate (32% Mn), 0.06% vitamin E premix (500,000 IU/kg), 0.05% copper sulfate, 0.01% selenium premix (0.99% Se), 0.007% ethylenediamine dihydriodide (EDDI) premix (11.4% EDDI), and 0.004% vitamin A (650,000 IU/g).

Sweet Bran (Cargill Corn Milling; Blair, NE).

Nutrient analysis conducted by SDK Laboratories (Hutchinson, KS).

Net energy of maintenance (NEm) and net energy of gain (NEg) were calculated using NASEM (2016) values of diet ingredients.

Feed bunks were assessed twice daily to determine the effects of bunk space allotment on rate of feed consumption. Feed bunks were evaluated 3 h (i.e., at 1000 h) and 6 h (i.e., at 1300 h) postfeeding using the feed bunk scoring system adapted from Boyles et al., (2003; Table 2). Briefly, feed bunks were assigned a score of 1–6 based on feed remaining in the bunk. A score of 1 indicated no feed residue remained while a score of 6 indicated greater than 30% of feed delivered at 0700 h remained.

**Table 2:** Feed bunk scoring system^1^

Score	Bunk Description
1	Empty Bunk; no feed residue remaining
2	Empty Bunk; evidence of fine feed particles
3	A few feed clumps and fine feed particles in the bunk
4	<15% of feed in bunk
5	15%–30% of feed in bunk
6	>30% feed in the bunk

Adapted from Boyles et al. (2003)

At the completion of the receiving period, steers were individually weighed, blocked by bunk treatment, and randomly assigned to 1 of 18 pastures (22 ± 4.0 ha). The following day, calves were treated for internal (Valbazen, Zoetis; Parisippany, NJ) and external (Stand Guard, Elanco; Greenfield, Indiana) parasites and administered a growth-promoting implant (Ralgro, Merck Animal Health; Kenilworth, NJ). Steers were sorted by pasture, held in pens where bunk allotment was not limited, and fed the experimental diet at 2.0% of BW (dry matter basis). Calves were allotted to their respective pasture over the following 3 d (i.e., six pastures per day). Individual BW were measured immediately prior to turnout. Steers were grazed for 90 d from May to August at a targeted density of 280 kg live weight ˖ ha^-1^. At the completion of the grazing period, steers were gathered and individual BW were immediately measured.

### Calculations

Individual BW measured on days 0, 29, and 58 were used to determine average daily gain (ADG) and gain-to-feed ratio (G:F), using pen-level intakes. Within pen variation in ADG was determined by calculating the standard deviation of ADG for each pen during the receiving period. Individual BW measured on days 0 and 90 of the grazing season were used to calculate grazing ADG. In addition, overall BW gains were calculated as grazing day 90 weight – receiving day 0 weight.

### Statistical Analysis

All data were analyzed as a randomized block design using the MIXED procedure in SAS (PROC MIXED; SAS 9.4, SAS Inst., Inc, Cary, NC). For performance during the receiving period, class variables included treatment, pen, and block. Two truckloads of calves were in each block, with 14 pens per block. The model included a fixed effect of treatment and random effect of block. For grazing and overall performance, pasture was added as a random effect. Treatment effects were evaluated using orthogonal, polynomial contrasts. For bunk score data, class variables included treatment, pen, block, and day. The model included fixed effects for treatment, day, and treatment × day and a random effect of block. Day served as the repeated term and the subject was pen. The covariance structure was spatial power as determined by AIC and BIC fit statistics. When protected by a significant *F*-test (*P* ≤ 0.05), treatment means were separated using the method of Least Significant Difference.

## RESULTS AND DISCUSSION

### Receiving Performance

Body weights on days 29 and 58 of the receiving period did not differ (*P* > 0.49; Table 3) between bunk treatments. During the first 29 d, ADG increased linearly (*P* = 0.03) with increased bunk space; however, no differences in ADG were observed thereafter. In addition, dry matter intake (DMI; *P* = 0.34) or G:F (*P* = 0.39) did not differ between bunk treatments following the 58-d receiving period.

**Table 3.** Effects of bunk space allotment on performance of limit-fed growing calves during a 58-d receiving period

Item,	Treatment, cm					*P*-value^2^		
	25	38	51	64	SEM^1^	Lin	Quad	Cubic
No. of pens	7	7	7	7				
No of animals	96	97	95	97				
Body weight, kg								
Day 0	214	216	215	216	3.5	0.76	0.93	0.67
Day 29	238	241	243	243	3.8	0.16	0.50	0.92
Day 58	257	260	263	260	3.6	0.38	0.29	0.58
ADG, kg/d								
0–29	0.81	0.88	0.98	0.94	0.067	0.03	0.23	0.38
29–58	0.65	0.64	0.70	0.59	0.047	0.40	0.15	0.10
0–58	0.73	0.76	0.84	0.76	0.045	0.22	0.10	0.12
DMI, kg/d								
0–29	4.10	4.10	4.11	4.10	0.013	0.48	0.12	0.27
29–58	4.76	4.76	4.80	4.75	0.037	0.89	0.44	0.30
0–58	4.42	4.41	4.46	4.42	0.025	0.56	0.50	0.12
G:F, kg/kg								
0–29	0.09	0.10	0.11	0.10	0.022	0.14	0.36	0.68
29–58	0.06	0.06	0.07	0.06	0.007	0.52	0.46	0.25
0–58	0.07	0.08	0.09	0.08	0.005	0.13	0.32	0.16
Bunk Score								
1000 h	1.64	1.72	2.03	1.83	0.451	0.06	0.20	0.13
1300 h	1.02	1.01	1.02	1.01	0.056	0.80	0.75	0.21

Mixed-model standard error of the mean (SEM) associated with comparison of treatment main-effect means.

*P*-value associated with linear, quadratic, or cubic effects of bunk allotment.

Our results agree with previous research that demonstrated limit-fed diets with bunk allotments of 12.7–60 cm per calf did not impact growth performance during the growing or finishing periods (Zinn, 1989; Gunter et al.,1996). Lake (1986) reported bunk allotments of 23 or 30 cm of bunk per head did not impact performance of limit-fed heifers fed twice daily (i.e., first half of their daily feed allotment at initial feeding and then the second half 2 h later). In addition, Harrison and Oltjen (2021) indicated bunk allotments of 20 cm or 87 cm per calf did not impact final body weights, DMI, ADG, or G:F following an 84-d growing period when steers were fed twice daily using the slick bunk protocol. Steers in our experiment were limit-fed once daily and growth performance did not differ between steers allotted 25.4–63.5 cm of bunk per calf. Despite differences in feeding protocols between our experiment and previous work, it appears that bunk allotments greater than 25.4 cm per head do not improve performance of growing calves when limit-fed a high-energy diet once daily.

A potential concern associated with reduced bunk space in limit-fed diets is an increase in weight variation within pen. ADG standard deviation from days 0 to 29 responded quadratically (*P* = 0.05; Table 4) where ADG standard deviation tended to be greater for the 38.1-cm allotment and was greater for the 50.8-cm allotment compared with the 25.4-cm allotment (*P* = 0.07 and 0.04, respectively). Gunter et al. (1996) observed similar trends, where reduced bunk allotment was associated with a linear decrease in final body weight variation within pen. In addition, Zinn (1989) indicated that bunk allotments of 15–60 cm per calf in finishing calves did not impact variation in final body weights and ADG within pen. Taken together these data suggest that reduced bunk allotment does not increase variation in weight gain.

**Table 4.** Effects of bunk allotment on average daily gain standard deviation of growing steers limit-fed a high-energy corn, corn co-product diet during a 58-d receiving period

Standard Deviation, kg/d	Treatment, cm					*P*-value^2^		
	25	38	51	64	SEM^1^	Lin	Quad	Cubic
0–58	0.28	0.30	0.31	0.31	0.040	0.32	0.75	0.96
0–29	0.37	0.47	0.48	0.43	0.049	0.22	0.05	0.92
29–58	0.30	0.28	0.29	0.34	0.049	0.40	0.36	0.95

Mixed-model standard error of the mean (SEM) associated with comparison of treatment main-effect means.

*P*-value associated with linear, quadratic, or cubic effects of bunk allotment

### Bunk Score

Bunks were evaluated daily at 1000 h and 1300 h to determine the impact of bunk allotment on rate of feed consumption. Bunk score at 1000 h tended to decrease linearly (*P* = 0.06; Table 3) with reduced bunk allotment. Reduced bunk score was interpreted to suggest that decreasing bunk allotment may result in more rapid feed consumption. Conversely, bunk score at 1300 h did not differ (*P* = 0.63; Table 3) between treatments. Bunk scores of 1.01–1.02 at 1300 h indicated that feed was consumed within 6 h of feed delivery. Schmidt et al. (2005) observed similar trends in feed consumption when evaluating the effects of feed restriction on growth performance of finishing beef steers. Steers restricted to 80% of ad libitum intake had a bunk score of 1.29 7 h after feed delivery. These data were interpreted to suggest that limit-fed diets used in these experiments were consumed within 6–7 h of feed delivery and a reduction in bunk allotment may increase rate of feed consumption.

### Grazing and Overall Performance

Body weights did not differ (*P* = 0.55; Table 5) at the beginning of the grazing period. ADGs during the grazing season increased linearly with reduced (*P* < 0.01; orthogonal polynomial contrast) bunk allotment; however, final body weights at the completion of the grazing period did not differ (*P* = 0.91; treatment main effect) between treatments. ADGs in our experiment were 1.10, 1.09, 1.04, and 1.05 kg ˖ calf^-1^ for 25.4-cm, 38.1-cm, 50.8-cm, or 63.5-cm bunk allotments, respectively.

**Table 5.** Effects of bunk allotment during the receiving period on subsequent growth performance during a 90-d grazing season and overall performance

	Treatment, cm						*P*-value^2^	
Item,	25	38	51	64	SEM^1^	Lin	Quad	Cubic
Body Weight,								
Day 0	273	277	279	278	4.75	0.25	0.38	1.0
Day 90	373	376	374	373	4.68	0.80	0.54	0.75
ADG, kg/d^3^								
Day 0–90	1.10	1.09	1.04	1.02	0.028	<0.01	0.99	0.39
Overall BW gain^4^								
Total gain, kg	159	160	159	156	3.2	0.34	0.38	0.96

Mixed-model standard error of the mean (SEM) associated with comparison of treatment main-effect means.

*P*-value associated with linear, quadratic, or cubic effects of bunk allotment.

Calculated as [(grazing day 90 weight – grazing day 0 weight) ÷ 90].

Calculated as (grazing day 90 weight – receiving day 0 weight).

The cause of the linear increase in ADG that resulted from reduced bunk allotment is unclear. Horton and Holmes (1978) evaluated the effects of feed restriction during a 20-wk period on subsequent growth performance during the grazing period. During the first 8-wk of the grazing season in that experiment, BW gains and DMI were greater in calves fed to gain 0.22 kg per day compared with calves fed to gain 0.58 kg per day. Wanyoike and Holmes (1981) fed 36 Friesian and Friesian crossbred steers at two growth rates (i.e., 0.5 or 1.08 kg per day) for a 12-wk period. Following the feeding period, steers were grazed on perennial ryegrass pasture. Body weight gains during the grazing period were greater in steers fed to gain 0.5 kg per day compared with steers fed to gain 1.08 kg per day. In addition, calves fed at a modest rate of gain consumed 12% more forage compared with calves fed at a more aggressive high rate of gain.

Lawrence and Pearce (1964) observed similar effects in weight compensation when feeding calves at high, medium, or low rates of gain (i.e., 0.73, 0.22, or 0.01 kg per day) for a 168-d period. During a subsequent 5-mo grazing period, total BW gains were 1.20, 0.98, and 0.54 kg per day for calves fed at low, medium, or high rates of gain, respectively. Although BW prior to grazing did not differ statistically in our experiment, BW decreased numerically with reduced bunk allotment. Calves assigned smaller bunk allotments during the receiving period may have experienced a small degree of body weight compensation during the grazing season. Reducing bunk allotment during the receiving period could have resulted in greater forage intake and improved ADG during the grazing season; however, overall total BW and ADG following the 58-d receiving period and the 90-d grazing season did not differ (*P* > 0.57) between bunk space treatments.

## CONCLUSIONS

These data suggest that bunk allotments of 25.4–63.5 cm of bunk per head had minimal impact on growth performance of limit-fed growing calves during a 58-d receiving period. Reduced bunk allotment tended to increase rate of feed consumption and reduce weight gain variation early in the feeding period. In addition, reduced bunk space during the receiving period was associated with increased ADG during the subsequent 90-d grazing season; however, final BW and overall BW gains following the receiving period and grazing season did not differ between bunk treatments. Overall, it appeared bunk allotments of 25.4–63.5 cm per calf were adequate for maintaining growth performance of growing steers limit-fed a high-energy corn and corn co-product diet. Under limit-feeding conditions, bunk allotments of 25.4 cm per calf may be used maximize pen capacity without reducing performance during the growing period.
